# Phlorotannin and Pigment Content of Native Canopy-Forming Sargassaceae Species Living in Intertidal Rockpools in Brittany (France): Any Relationship with Their Vertical Distribution and Phenology?

**DOI:** 10.3390/md19090504

**Published:** 2021-09-04

**Authors:** Camille Jégou, Solène Connan, Isabelle Bihannic, Stéphane Cérantola, Fabienne Guérard, Valérie Stiger-Pouvreau

**Affiliations:** 1 Laboratoire de Biotechnologie et Chimie Marine (LBCM) EA 3884, Université de Brest, 6 Rue de l’université, F-29334 Quimper, France; camille.jegou@univ-brest.fr; 2 Laboratoire des Sciences de l’Environnement (LEMAR) UMR 6539, Université de Brest, CNRS, IRD, Ifremer, F-29280 Plouzane, France; solene.connan@univ-brest.fr (S.C.); isabelle.bihannic@univ-brest.fr (I.B.); Fabienne.Guerard@univ-brest.fr (F.G.); 3 Service Commun de RMN-RPE, Université de Brest, F-29200 Brest, France; stephane.cerantola@univ-brest.fr

**Keywords:** phenolic compounds, phlorotannins, photoprotective pigments, phenolic compounds structure, bathymetry, tide pool, intertidal algal distribution

## Abstract

Five native Sargassaceae species from Brittany (France) living in rockpools were surveyed over time to investigate photoprotective strategies according to their tidal position. We gave evidences for the existence of a species distribution between pools along the shore, with the most dense and smallest individuals in the highest pools. Pigment contents were higher in lower pools, suggesting a photo-adaptive process by which the decreasing light irradiance toward the low shore was compensated by a high production of pigments to ensure efficient photosynthesis. Conversely, no xanthophyll cycle-related photoprotective mechanism was highlighted because high levels of zeaxanthin rarely occurred in the upper shore. Phlorotannins were not involved in photoprotection either; only some lower-shore species exhibited a seasonal trend in phlorotannin levels. The structural complexity of phlorotannins appears more to be a taxonomic than an ecological feature: *Ericaria* produced simple phloroglucinol while *Cystoseira* and *Gongolaria* species exhibited polymers. Consequently, tide pools could be considered as light-protected areas on the intertidal zone, in comparison with the exposed emerged substrata where photoprotective mechanisms are essential.

## 1. Introduction

In recent years, more attention has been paid to specific habitats on rocky shores known as “tide pools”. These biotopes represent isolated puddles found all over the rocky substrate on the intertidal zone, naturally retaining water at low tide. Although pools are sometimes not considered as intertidal habitats [1] because of the absence of emersion periods, they cannot be classified as subtidal areas either, but rather as a refuge area for both intertidal and subtidal organisms which will remain immersed [2]. Nevertheless, as for the emerged substrata, physical and chemical variations of the water in pools have been analyzed according to their tidal level [3,4,5,6,7]. Actually, the composition in macroalgae found in tide pools can vary according to the pool position on the shore [5,8], such as on emerged substrata, or not [9,10]. Tide pools were once considered unstable systems under the dependence of sudden disturbance [11], but a contradictory conclusion was brought out by Astles [12]. In a whole, little is known about ecology in such pools, compared to the extensive literature concerning the emerged intertidal zone, and only fragmental or sometimes contradictory information has been evidenced. For the coast of Brittany (France), a pattern of seaweed distribution depending on the location of the pool along the sea shore has been introduced by Cabioc’h et al. [13], and a bathymetric distribution inside some pools, analog to the algal belts observed along the sea shore, has been evidenced [8]. A particularly important missing piece of data is, in our opinion, a monitoring about typical or even exclusive tide pool species, concerning both distribution patterns and spatial and temporal variations of their populations.

Sargassaceae species, altogether with Fucaceae ones, are among the most abundant brown macroalgae occurring along the coasts of Brittany [14,15]. In Europe, main native species from the family Sargassaceae are typically located in intertidal rockpools and/or in the subtidal zone, especially those from the genera *Cystoseira*, *Ericaria* and *Gongolaria*. These genera are quite common in Brittany, and they occupy pools located at nearly all tide levels. Actually, the genera *Cystoseira*, *Ericaria* and *Gongolaria*, settling in the subtidal zone and/or in rockpools of the intertidal zone, have been subject of a few studies on their ecology in Brittany, in comparison with the numerous data available for the surrounding coralline algae [7,16] and *Bifurcaria/Fucus/Sargassum* species [14,17,18,19]. Within this Sargassaceae family, different native genera are present in intertidal rockpools from Brittany and constitute large biomass and canopies [13,20]: *Cystoseira fœniculacea*, *C. humilis*, *Ericaria selaginoides* (previously known as *Cystoseira tamariscifolia*), *Gongolaria baccata* and *G. nodicaulis* (previously *C. baccata* and *C. nodicaulis*). According to Cabioc’h et al. [13], *Cystoseira humilis* would be strictly located on the intertidal zone, in exposed pools. In Brittany (Atlantic Ocean), this species exclusively occurs in tide pools, contrary to the Mediterranean Sea, where it can extend down to the subtidal zone [21,22] and references therein. These species can represent an important biomass, making it easily suitable for such a study. Each of the five species has its own pattern of distribution on the shore [20]; consequently, the populations have to face different environmental conditions, in particular tidal variations followed by emersion of rockpools, and then variability in the quantity and quality of light, and seawater salinity and temperature. As they colonize tide pools, these native Sargassaceae species do not face desiccation trouble. However, there are several abiotic factors varying in pools along the shore [4,5] and especially in Brittany [16,20]. For example, macroalgae in tide pools are submitted to variations in temperature, even if the range of variation is narrower than on the emerged substrata due to the persistence of water in pools. In addition, the light irradiance can dramatically fluctuate in pools depending on their location on the intertidal zone. In the uppermost pools, the intense photosynthetically active and ultraviolet radiations can disturb the development of macroalgae. In this way, different photo-adaptive strategies could be expected for the species, according to their distribution on the shore. Among strategies developed by brown macroalgae face to light, two kinds of compounds are implicated in the tolerance to excessive light irradiance: carotenoids and phlorotannins [23].

Carotenoids are the main class of photoprotective compounds, consisting in xanthophylls and carotenes. Under high light irradiance, the thermal dissipation of excessive energy is ensured in brown macroalgae by the xanthophyll cycle [24,25]. It involves two de-epoxidations of the photosynthetic violaxanthin which is then transformed in the photoprotective zeaxanthin which can dissipate excessive energy [24]. These two pigments can also be involved in the detoxification of reactive oxygen species formed during photosynthesis, with other carotenoids such as fucoxanthin and β-carotene [26,27]. The xanthophyll cycle has been unequivocally evidenced as a major photoprotective mechanism of the brown macroalgae on the intertidal zone. Gévaert et al. [28,29] showed that the kelp *Saccharina latissima* (located on the low intertidal zone) used the xanthophyll cycle to dissipate energy at low tide. Furthermore, Harker et al. [30] proved that *Pelvetia canaliculata* had a greater xanthophyll pool than *S. latissima*. This could partially explain the aptitude of the alga to settle very high on the shore. Uhrmacher et al. [31] revealed that photoinhibition was realized by the xanthophyll cycle in the brown macroalga *Dictyota dichotoma*.

On the other side, phlorotannins belongs to a family of biomolecules typical of brown macroalgae, derived from the polymerization of phloroglucinol (1,3,5-trihydroxybenzene, 126 Da). They are thus part of the group of polyphenols. These phlorotannins are involved in both the primary and secondary metabolism of Phaeophyceae [32,33,34]. Phlorotannins are produced by condensation of malonate and acetate under the action of a polyketide synthase (PKS) enzyme [35,36]. The final structure of each phlorotannin depends on three parameters: (1) The degree of polymerization, which indicates the number of phloroglucinol units assembled to form the molecule in question. This degree is extremely variable, from oligomers up to 650 kDa polymers [37]. (2) The type of polymerization, which reflects the way the phloroglucinol units are linked. Linkages occur in three different ways, according to [38,39,40]: Aryl-Aryl (C-C) in the case of Fucols, Diaryl-Ether (C-O-C) in the case of Phlorethols and (Iso) Fuhalols, Dibenzo-dioxin (C-O-C) in the case of Eckols and Carmalols, reviewed by Stiger-Pouvreau et al. [41] for Sargassaceae species from Brittany. (3) The presence of substitutions on the rings, including halogen [42] and sulphate [43]. Numerous studies have investigated their ecological and cellular roles. Phlorotannins are notably thought to have a sunscreen action, as demonstrated in *Halidrys siliquosa* [44]. Lalegerie et al. [45] reviewed the ecological and ecophysiological roles of phenolic compounds from red and brown seaweeds, and Gager et al. [46] their biological activities.

Our study was designed to evidence the distinctive distribution of the main native canopy-forming genera of Sargassaceae (Phaeophyceae, Fucales), i.e., *Cystoseira*, *Ericaria* and *Gongolaria*, occurring in tide pools from Brittany, according to their position on the shore. For this purpose, we have compiled three different studies (from winter 2009 to spring 2011) in order to investigate the involvement of pigments and phlorotannins (composition and type) on the phenology and distribution of the considered species. We have also analyzed the distribution of the five species inhabiting tide pools in Brittany, at three tidal heights, and surveyed the spatial and temporal variations of some biological and chemical (phlorotannins and pigments) characteristics of their populations. Our purpose was to verify whether the photosynthetic capacity and the photoprotective mechanisms are specific characters, hence explaining the distribution of the species, or if they can be modulated within populations according to the tidal height where individuals settle. Finally, we present the type of phlorotannins produced by each species, as protective compounds for their persistence in intertidal rockpools.

## 2. Results

### 2.1. Distribution of the Five Species along the Intertidal Rockyshore

**Figure 1:**
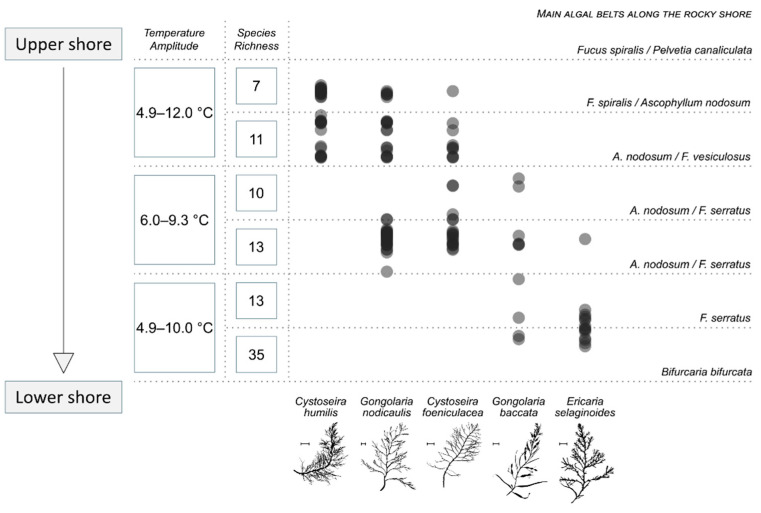
Compiled distribution of individuals from the five species of *Cystoseira*, *Gongolaria* and *Ericaria* along the intertidal zone at Penmarc’h (Brittany) over a one-year survey, from spring 2 to spring 3. The presence of individuals in one quadrat on a precise position along the transect is indicated by one semi-transparent grey circle. Correspondence between levels and Fucales belts is given (right side), as well as the observed macroalgal species richness, temperature amplitude of rockpools for 6 h on a winter day (left side). Profiles of young apical branches of the five studied species from seaweed specimens are also drawn; scale bar: 1 cm.

The transect showed a typical distribution pattern of the five species. On Figure 1 are plotted the position of the quadrats where individuals of each species were found. The species richness of each pool is also presented, as well as the correspondence of the level on the shore with the classical algal belts present on intertidal temperate rocky shores. In summary, the species succession from the upper to the lower shore was as follows: *Cystoseira humilis, Gongolaria nodicaulis, C. fœniculacea, G. baccata*, and *Ericaria selaginoides*, with a decrease in rockpool species richness in the upper shore (Figure 1).

*Cystoseira humilis* and *E. selaginoides* had a narrow distribution on the shore. *Cystoseira humilis* was strictly settled in the upper pools, just underneath the *Fucus spiralis* belt. The macroalgal species richness associated with the rockpools dominated by *C. humilis* was low (seven species) (Figure 1). *Ericaria selaginoides* was located roughly from the *F. serratus* belt to the *Bifurcaria bifurcata* belt, in the very lower shore. In such pools, the high species richness (from 13 to 35 different taxa) is mainly due to the occurrence of red macroalgae. *Gongolaria baccata*, *C. fœniculacea* and *G. nodicaulis* had a wider distribution and colonized several pools along the rocky shore. The first had a scattered distribution under the *Ascophyllum nodosum*/*F. vesiculosus* belt (species richness from 10 to 35), and the two others were located on the mid shore between *F. spiralis* and *F. serratus* belts (species richness from 7 to 13), where brown, green and red macroalgae coexist. The salinity of each pool was quite stable and around 35.3 over the 6-h period of measurement during the one-year survey (from spring 2 to spring 3). Conversely, temperature varied with seasons, and interestingly, during the day, in ranges depending on the considered pool. A monitoring of the variability of the seawater temperature was made during a winter day as presented in Figure 1: a range of nearly 7 °C was noted for the upper rockpools, while on the lower shore the range was of nearly 5 °C. The middle part of the shore represented an area where only a 3 °C difference was observed between the coldest and the warmest temperature

### 2.2. Phenological Variables

On the Table 1, the density varied significantly (*p* < 0.001), with maximal values for the populations of *C. humilis* especially in summer; then the density decreased for *C. fœniculacea*, *G. nodicaulis* and *G. baccata*, and was minimal for *E. selaginoides*. The percentage cover is maximal in lower shore species, reaching 90% and 91.67% for *G. baccata* and *E. selaginoides*, respectively. Regarding the maturity index observed in the quadrats, it appeared maximal for *G. nodicaulis* and *G. baccata* (with no statistical difference between them), and the individuals of the other species less frequently bore receptacles (Table 1).

The *G. baccata* population turned out to be only made of mature individuals, no matter the season. The individual length significantly differed between species (*p* < 0.001), and from the smallest to the tallest, the order was *Cystoseira humilis*, *G. nodicaulis*/*C. fœniculacea*, *E. selaginoides* and the largest *G. baccata*. The maximal lengths of individuals were found in populations where the density remained low (i.e., *G. baccata* and *E. selaginoides*), and at the opposite, *C. humilis* which could present the densest population was characterized by the smallest individuals (Table 1). *Cystoseira fœniculacea* and *G. nodicaulis* were intermediate; individuals of these species were neither small nor tall, and a medium density range was observed in the quadrats. Site, season and their interaction affected the length in the *C. humilis* populations, and fertile individuals could be observed anytime of the year. *Cystoseira fœniculacea* and *G. nodicaulis* thalli appeared taller than *C. humilis* (Table 1). *Gongolaria baccata* and *E. selaginoides* were characterized by the tallest thalli and the lowest density. The length of both species was affected by season, but only the latter has a marked seasonal growth, beginning in late winter, and ending around the summer when receptacles are fully developed, in accordance with the observations on the maturity index (Table 1).

### 2.3. Pigment Composition and Inter-Species Variability

**Figure 2:**
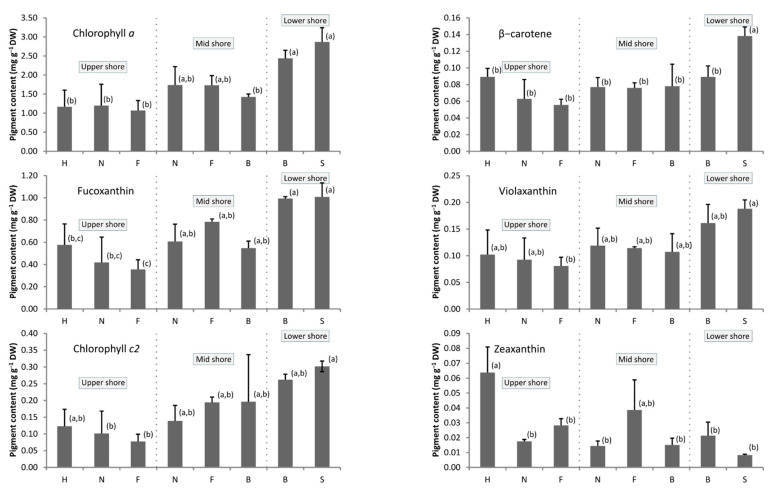
Pigment contents (mg·g^−1^ algal DW) in spring 2011 (=spring 3) in the five native Sargassaceae species from Brittany (France) according to their position on the shore. *Ericaria selaginoides* “S”, *Gongolaria baccata* “B”, *Cystoseira fœniculacea* “F”, *G. nodicaulis* “N” and *C. humilis* “H”. a, b: for each pigment analysis, values sharing a common letter were not statistically different according to Tukey’s HSD test. The scale for each graph is not similar. Mean + sd; *n* = 6 for each species and position along the shore.

In all samples, the major pigments were chlorophyll *a*, fucoxanthin, and chlorophyll *c2* (Figure 2). Their maximal mean contents reached 2.8, 1.0 and 0.3 mg·g^−1^ algal DW, respectively. Maximal values were always observed in *E. selaginoides*. Violaxanthin, β-carotene and zeaxanthin contents were characterized by lower values, under 0.2, 0.14 and 0.08 mg·g^−1^ DW, respectively. All pigment contents depended on the considered species (Table S1, *p* < 0.05). The “tidal height” effect could be evidenced only for chlorophyll *a*, fucoxanthin and violaxanthin. There was no significant interaction between the two factors, “species” and “tidal heights”, which means that the effect of tidal height on chlorophyll *a*, fucoxanthin and violaxanthin content did not depend on the species, and vice versa. Hence, for these three pigments, the content globally increased from the upper to the lower intertidal pools (HSD, *p* < 0.05; Figure 2). The low shore species *E. selaginoides* and the low/mid shore *G. baccata* were characterized by high content for all pigments, except zeaxanthin for which they had minimal values (Figure 2, HSD test: *p* < 0.05).

Conversely, the upper alga *C. humilis* had the maximal zeaxanthin level and low contents for the other pigments. Even if this was not always supported by statistical analyses, the levels of chlorophyll *a*, fucoxanthin, chlorophyll *c2*, violaxanthin and β-carotene decreased for each species, from one location on the shore to a lower one (Figure 2). We evidenced a similar evolution of several pigments within species and tidal height using Pearson’s correlation test (Table S2): except zeaxanthin, all pigments were highly correlated (0.71 < ρ <0.90; *p* < 0.001) and thus evolved in the same manner through species and tidal height. The results for zeaxanthin are less clear; its highest levels occurred in the uppermost pools with *C. humilis* (around 0.06 mg.g^−1^ DW), but no statistical difference was observed in the rest of the dataset (Tukey’s HSD test, Figure 2).

### 2.4. Inter-Species and Inter-Seasonal Variabilities of Phenolic Contents

**Figure 3:**
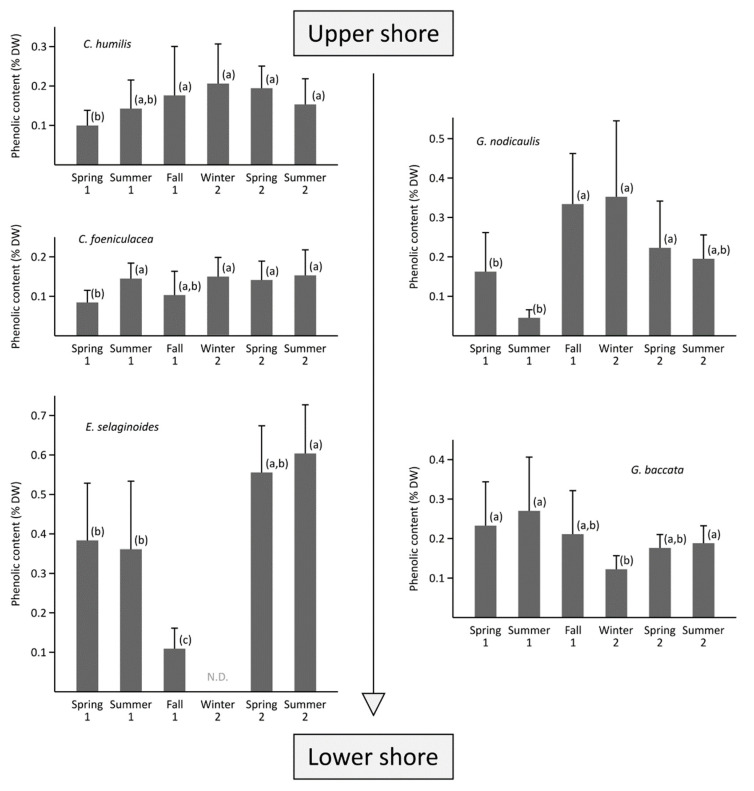
Evolution of the phenolic contents (% DW), in phloroglucinol equivalent, of semi-purified extracts from the five species of *Cystoseira*, *Gongolaria* and *Ericaria*. a, b, c: two same letters found in a species during two seasons indicate that their contents are not statistically different, after ANOVA. N.D.: no data, as *E. selaginoides* was represented only by basal parts with no lateral. Mean ± standard deviation; *n* = 18 for each studied species and each season.

The average phenolic compound (PC) content varied according to the five species and the sampling season. The five species showed significantly different PC contents (Figure 3, Kruskal–Wallis, *p* < 0.001): *Ericaria selaginoides* had the highest mean PC content (0.42 ± 0.21% DW), followed by *G. nodicaulis* and *G. baccata* with, in average, 0.25 ± 0.16% DW and 0.21 ± 0.10% DW, respectively. The PC content of *C. humilis* (0.19 ± 0.09% DW) was not significantly different than *G. baccata* one. Finally, *C. fœniculacea* (0.13 ± 0.06% DW) contained the lowest PC level. Among the five species studied, two groups can be distinguished (Figure 3): a first group of two species with no significant seasonal trend in phenolic content (*C. fœniculacea* and *C. humilis*), with PC contents ranging from 0.08 ± 0.03% DW (spring 1) to 0.15 ± 0.06% DW (summer 2) and 0.10 ± 0.04% DW (spring 1) and 0.21 ± 0.01% DW (winter 2), respectively.

In contrast, the three other species showed marked seasonal variation: *Gongolaria baccata* showed a decrease in PC content from 0.27 ± 0.14% DW in summer 1 to 0.12 ± 0.03% DW in winter 2 (Figure 3). Conversely, the PC levels of *G. nodicaulis* oscillated between a maximum (0.35 ± 0.19% DW) from autumn 1 to winter 2, and a minimum observed before and after this period (0.04 ± 0.01% DW in summer 1). *Ericaria selaginoides* showed averaged phenolic content from spring to summer 1 (0.38 ± 0.15% DW). Then, its PC content decreased to reach its minimum in autumn 1 (0.11 ± 0.05% DW). In winter 2, due to the senescence of the species (only the basal parts remained), no sample has been collected. In spring 2, high levels can be observed (Figure 3), which increased again to a maximum in the following summer (0.60 ± 0.13% DW).

Considering the variability of phenolic content within a year, the three species from the upper shore, *Cystoseira humilis*, *C. fœniculacea* and *Gongolaria nodicaulis*, have higher levels in winter and lower levels in spring/summer, while the two lower species, *G. baccata* and *Ericaria selaginoides*, have higher levels in spring/summer and lower levels in winter.

### 2.5. Purification of Phlorotannins and NMR Analyses

**Figure 4:**
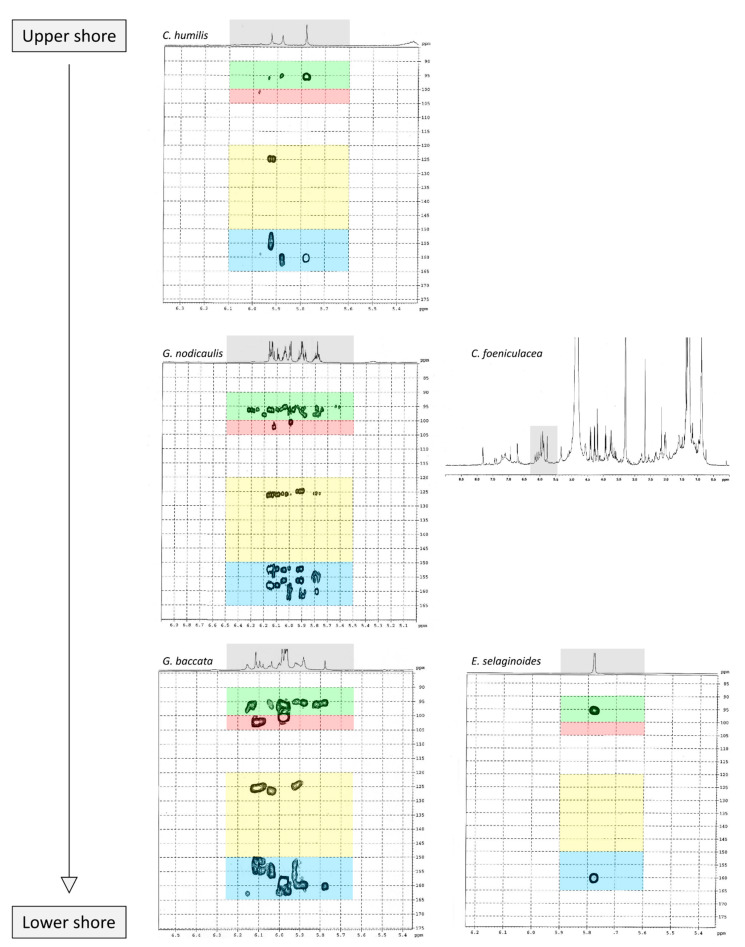
^1^H-^13^C HMBC NMR spectra of the purified fractions (solvent: MeOD). Horizontal axis: ^1^H dimension; vertical axis: ^13^C dimension. Only a ^1^H spectrum is given for *C. fœniculacea*. Grey box: phlorotannins area (circa 5.7–6.5 ppm in ^1^H dimension); green (90–100 ppm in ^13^C dimension): methine zone; red (100–105 ppm): carbons involved in an aryl-aryl bond between two phloroglucinol units; yellow (120–150 ppm): diaryl-ether bond; blue (150–165 ppm): carbons with phenol function.

Figure 4 shows 2D ^1^H-^13^C HMBC and 1D ^1^H NMR spectra of the purified fractions for the five *Cystoseira*, *Ericaria* and *Gongolaria* species. All purified fractions presented different aromatic signals in the chemical shift zone between 5.7 and 6.5 ppm, attesting to the potential presence of phlorotannins (Figure 4). Moreover, these signals were different depending on the species. We can also highlight the presence of aliphatic signals of non-negligible intensity in the fractions of *G. baccata*, *C. fœniculacea*, *C. humilis* and *G. nodicaulis*, which attests the presence of compounds other than phlorotannins. On the other hand, the purified fraction of *E. selaginoides* indicated the overwhelming presence of an aromatic compound. It appears as a singlet at 5.78 ppm in deuterated methanol (Figure 4), indicating a single type of proton in the molecule, all chemically equivalent. This signal is due to phloroglucinol, the monomer at the origin of phlorotannins. By observing the diversity of phlorotannins between each species (form and number of peaks around 6 ppm, Figure 4), one should note that these molecules, as polymers of phloroglucinol, share a structural characteristic: among all these molecules, the protons visible in ^1^H NMR in MeOD are those of the methine groups (C-H of the aromatic rings).

Following the results obtained on ^1^H NMR spectra, a 2D ^1^H-^13^C NMR analysis of the purified fractions was carried out for four of the five species studied. Indeed, the low phenolic content in *C. fœniculacea*, associated with the chemical instability of the purified fraction, did not allow us to present a Heteronuclear Multiple Bond Correlation (HMBC) spectrum for this species.

The HMBC spectrum (Figure 4) of *Gongolaria nodicaulis* showed a multitude of signals on the ^1^H dimension, which correlated with methine-like carbons (C-H of the aromatic rings), phenol-like carbons (C-OH), rare carbons involved in aryl-aryl ring bonds (C-C) and a majority of carbons involved in diaryl-ether ring bonds (C-O-C). In *G. baccata*, a smaller number of correlation spots were observed (Figure 4). We can thus note, opposite the signal at 5.98 ppm in ^1^H dimension, a large correlation spot at 101 ppm in ^13^C dimension. However, there is no correlation spot between 120 and 150 ppm, suggesting the presence of a large linear polymer of the fucol type. Indeed, lateral branching would generate small variations in chemical shifts in the ^1^H dimension, and a less well-defined correlation spot would then be observed. By similar reasoning, we can detect a phlorotannin of the phlorethol type (δ = 5.92 ppm in ^1^H dimension), and a fucophlorethol (δ = 6.10 ppm). There was also a signal at δ = 5.78 ppm (isolated signal in ^1^H dimension), which correlated only with methines and phenolic carbons (Figure 4). This compound, which translated to a singlet in ^1^H dimension, present in the pure state in the purified fraction of *E. selaginoides* (Figure 4), was also the major compound in the purified fraction of *C. humilis* (Figure 4) and identified as phloroglucinol. In *C. humilis*, we also found a linear or slightly branched phlorethol-type compound (δ = 5.92 ppm in ^1^H dimension), as well as another compound at δ = 5.88 ppm (^1^H dimension), which showed neither an aryl-aryl bond nor a diaryl-ether bond (Figure 4). This compound, which “looked like” phloroglucinol (^1^H δ = 5.78 ppm), differed by a doubling of the C-OH correlation spot around 160–163 ppm in ^13^C dimension. In *E. selaginoides*, the purified fraction showed only one major compound identified as phloroglucinol.

In summary, Table 2 presents the type of phlorotannins that could be identified in the five species of concern. The upper species *C. humilis* produced phloroglucinol and phlorethols. The median species *G. nodicaulis* did not produce phloroglucinol but three types of phlorotannins: fucols, phlorethols and fucophlorethols.

Both lower species, *G. baccata* and *E. selaginoides* produced different types of phlorotannins: *E. selaginoides* synthetized only the monomer phloroglucinol, conversely to *G. baccata* which was able to produce phloroglucinol (traces), and the three types of polymers, fucols, phlorethols and fucophlorethols (Table 2).

## 3. Discussion

This study focuses on the zonation pattern of five native canopy-forming Sargassaceae species occupying intertidal rockpools in Brittany (France) and investigates a potential relationship between tidal height, chemical content (pigments, phlorotannins) and phenological variables. Using field data, we specify the distribution of each species on the foreshore of Penmarc’h, which is generalizable to the Brittany region. Thus, *Gongolaria baccata* and *Ericaria selaginoides* live at the limit of low tide levels; *G. nodicaulis* and *Cystoseira fœniculacea* are at the mid-tide level, and *C. humilis* at the high tide level.

### 3.1. Photo-Adaptation along the Shore but No Photoprotective Pigments in the Upper Shore

Chlorophyll *a*, fucoxanthin and chlorophyll *c*2 are naturally the main pigments for all species at all tidal levels, as they are involved in photosynthesis [47]. Similar results were obtained from two brown macroalgae, *Pelvetia canaliculata* [48] and *Saccharina latissima* [49]. We also observed minimal photosynthetic pigments contents for macroalgae settled in the upper shore, and maximum in the lower shore (Figure 2). For example, *E. selaginoides* produced more pigments than *C. humilis* (except zeaxanthin), and *G. baccata* produced more chlorophyll when living in the lower intertidal zone than in the mid-zone. This increase in pigment production in species living in lower levels counterbalanced the reduced level of irradiance in this lower intertidal zone, as already suggested [50,51,52]. Furthermore, on the higher shore, high levels of irradiance or UV radiations could reduce pigment levels, as experimentally evidenced for *Fucus vesiculosus* [53]. On the other hand, no clear pattern could be observed looking at the evolution of the photoprotective zeaxanthin levels on different tidal heights (Figure 2). Only differences between species were observed, due to high levels occurring in *Cystoseira humilis* and in the mid *C. fœniculacea* populations. However, low zeaxanthin levels were determined in the upper individuals of *G. nodicaulis* and *C. fœniculacea* (Figure 2). The literature reports indicated that zeaxanthin production was deeply implicated in photoprotective mechanisms through the xanthophyll cycle via the double de-epoxidations of violaxanthin as it was demonstrated in the lower shore *Saccharina latissima* [28,29] and in the upper shore *Pelvetia canaliculata* [30]. Moreover, very high quantities of violaxanthin occurring in the last species were considered as a potential zeaxanthin pool that would be used as a way to dissipate energy in case of extreme light conditions. In this study, neither high content of violaxanthin nor high quantity of zeaxanthin could characterize the macroalgae settled in the upper shore. Apparently, there is no need for particular photoprotective mechanisms in these species living in the upper pools.

Consequently, our study suggests that photo-adaptation exists in rockpools. Depending on the tidal height, the species receive different amounts of light and regulate their pigment synthesis in order to maintain a good photosynthetic activity. In opposition, the xanthophyll cycle does not seem to be particularly active in the upper pools, indicating that light irradiance is not high enough to over-activate photosynthesis. The thin layer of water that persists in rockpools at low tide may represent an efficient screen against excessive light irradiance. Further investigations should be undertaken particularly during the sunniest days of summer to state whether photoprotective mechanisms could be employed when the algae are submitted to exceptional light irradiance. Measuring the variations of the xanthophyll pigments in situ could also be a successful approach, as experienced by Gévaert et al. [29].

### 3.2. What Are the Drivers of the Variability of Phenolic Content in the Five Species?

To our knowledge, this study is the first to characterize the seasonal variability of phlorotannins within the genera *Cystoseira*, *Ericaria* and *Gongolaria* from Brittany. In this study, these phenolic compounds were first purified by a liquid–liquid ethyl acetate (EA) extraction before being quantified by the Folin-Ciocalteu method; this purification step is commonly used to isolate phlorotannins [41,44,54,55] and more generally phenolic compounds from marine plants [56,57].

**Interspecific variability.** Our study demonstrates an interspecific variability in phenolic contents between the five species. Over the monitoring at Penmarc’h, we were able to determine that the levels of phenolic compounds were higher in *E. selaginoides*, *G. nodicaulis* and *G. baccata*, while *C. fœniculacea* and *C. humilis* had lower levels (below 0.2% DW) (Figure 3). Among the different roles attributed to phlorotannins, a photoprotective role was considered by Pavia et al. [58,59]. These authors showed an induction of phlorotannin production in *Ascophyllum nodosum* following the exposure of thalli to high doses of UV-B light. Due to the numerous aromatic rings constituting these molecules, they strongly absorb certain UV radiation.

Related to the distribution of each species along the intertidal rocky shore, *C. humilis* received the greatest amount of UV radiation because the shallow pools in which it settled are only submerged for a short time during a tidal cycle. However, its phenolic content was much lower than *E. selaginoides*, which was less exposed to strong radiation due to its low position on the foreshore. It is therefore impossible to establish a direct relationship between species distribution on the foreshore and phenolic content. However, phlorotannins are also known to be exuded into the surrounding environment, as already demonstrated [60,61]. Connan et al. [14] hypothesized this exudation to explain the lower content in the upper *Pelvetia canaliculata* compared to the high phenolic content determined in the median *Ascophyllum nodosum*. If the phlorotannins of *C. humilis* have an anti-UV action, perhaps they were rather exuded into the rockpool. The levels observed in vivo are probably dependent on other factors, like the diversity associated with each rockpool. The levels of phenolic compounds could also reflect the taxonomic group of the algal species. Indeed, the genus *Ericaria* (0.42% DW) produced more phenolic compounds than *Gongolaria* (0.21–0.25% DW) followed by *Cystoseira* (0.13–0.19% DW). Our quantitative study of phlorotannins supports the idea that *C. fœniculacea* and *C. humilis* adopt a fundamentally different chemical defense strategy than *G. baccata*, *G. nodicaulis* and *E. selaginoides*.

**Seasonal variability.** The phenolic contents in *Cystoseira humilis* did not show a seasonal pattern (Figure 3). This result is not surprising; indeed, the population of this species was in dynamic equilibrium, and perpetually renewed (many recruits constituted the density in summertime). No seasonality was observed, not in the size of the individuals, the density or the presence of mature individuals. The same result was observed for *C. fœniculacea*, whose phenolic content did not vary significantly during the six studied seasons (Figure 3). On the contrary, this species developed seasonally, with individuals shedding their shoots in late spring, and developing progressively until late winter. Thus, phlorotannin levels in this species appeared to be independent of the phenological state of the individuals.

Although we did not determine a phenological cycle in *Gongolaria baccata*, whose individuals were mature throughout the year in Penmarc’h (South Finistère), we noted lower levels of phenolic compounds in winter, and higher levels in summer (Figure 3). Our results are contradictory to those established by Le Lann et al. [19], who observed higher levels during winter and summer from samples taken in Porsmeur (North Finistère). The origin of this difference may be due to the purification step that we included in our study, whereas Le Lann et al. [19] determined the polyphenols by the Folin–Ciocalteu method using crude extracts. Further studies are needed to determine the origin of the observed seasonal variability because here the relative stability of the population parameters contrasts with the seasonality of the phenolic contents. An environmental factor that does not influence the size, density or maturity of the individuals could explain these seasonal variations in phlorotannin concentrations, such as epiphytism or herbivore pressure.

Regarding *G. nodicaulis*, its phenolic content evolved in parallel with the phenology of the species: contents were minimal in spring/beginning of summer, when the branches started to fall naturally. It can thus be considered that a form of progressive senescence from spring onwards led to a decrease in concentrations, after which the branches were regenerated from the autumn onwards, with an increase in phenolic contents. Finally, in winter, some twigs began to detach, and levels decreased, heralding the resting period and lower levels observed in early summer (see results in Figure 3).

*Ericaria selaginoides* is characterized by even clearer seasonal variations (Figure 3). Indeed, the end of summer can be contrasted with the other times of the year: the phenolic levels were particularly low (0.1% DW) in autumn, in contrast to spring or summer when they exceeded 0.4% DW. Such as *G. nodicaulis*, twig loss in *E. selaginoides* was initiated during the summer, and was maximal at the end of summer (Table 1). Thus, the summer senescence of shoots is a plausible explanation for these particularly low phenolic levels. Our results are not inconsistent with the observations of Abdala-Díaz et al. [62] who showed a progressive increase in levels between spring/summer, followed by a decrease from summer to winter in Spain. The authors also showed that the levels of phenolic compounds are correlated with the level of light radiation, and they deduced a photoprotective role of these compounds. In our study, we observed a more drastic decrease in levels in summer, which was probably related to the senescence that occurred in this period. Unfortunately, Abdala-Díaz et al. [62] did not specify the phenology of *E. selaginoides* in Spain. This data would be particularly interesting to be able to compare the strategy of this species between French and Spanish populations.

Each species was characterized by its own seasonal dynamics of phlorotannin levels. In the case of *E. selaginoides* and *G. nodicaulis*, phlorotannin levels seemed to evolve in parallel with the phenology of the alga. These compounds were accumulated in the tissues before the maturity period, probably to protect the receptacles from herbivore grazing. Further investigations, extending the monitoring over several years and also measuring the exuded phenolic compounds in the rockpools, could shed some light on the ecological role of phlorotannins.

### 3.3. Do the Five Species Produced the Same Phlorotannins?

Our study demonstrates that the five species did not produce similar phlorotannins. *Ericaria selaginoides* produced the monomer phloroglucinol and not more complex phlorotannins, *Cystoseira humilis* produced phloroglucinol and phlorethol, *Gongolaria nodicaulis* produced three types of phlorotannins, fucol, phlorethol and fucophlorethol and finally, *G. baccata* was able to produce the three types of phlorotannins and the monomer (Table 2). The types of phlorotannins identified in both species of *Gongolaria* were essentially the same: fucols as well as phloroethols. The absence of signals at 145–150 ppm indicates that the extracts did not contain a fuhalol-type polymer [63]. This seems surprising because fuhalols have been characterized by the past in *G. baccata* [64] and in *G. nodicaulis* [65]. At the same time, small amounts of phloroglucinol were found in *G. baccata* and *C. humilis*. In *E. selaginoides*, large amounts of phloroglucinol were observed, but no traces of bifuhalol and diphlorethol, already identified in this species by Glombitza et al. [66]. According to the NMR data of diphlorethol and bifuhalol (also described in *Bifurcaria bifurcata* by Glombitza and Rösener [67]), the NMR signals of the aromatic protons of these compounds would be one doublet and one triplet, which are not visible on the ^1^H NMR spectrum of *E. selaginoides* (only a singulet; Figure 4).

According to the results of our study, phlorotannin types are not a relevant criterion from a chemotaxonomic point of view. Indeed, fucols and phloroethols are very common molecules among the species considered. However, phloroglucinol can be used as a chemotaxonomic marker for *E. selaginoides* because it is the only species that exclusively produces the monomer phloroglucinol. By HR-MAS NMR analyses, Jégou et al. [68] highlighted that *E. selaginoides* produced phloroglucinol in spring and summer until the beginning of autumn and then the species entered a dormancy-like phase during autumn and winter in Brittany. Using an innovative qNMR technique, authors were able to specifically quantify the intra-individual and seasonal variations of phloroglucinol [68]. Further experiments on *E. selaginoides* under controlled conditions should be carried out in order to determine which parameters (temperature, salinity, light radiation, nutrients, and/or herbivore pressure) induce the synthesis of phenolic compounds.

The diversity of phlorotannins within Sargassaceae species was already demonstrated by Stiger-Pouvreau et al. [41] on the eight species encountered in Brittany, including the five species studied here, together with *Bifurcaria bifurcata*, *Halidrys siliquosa* and *Sargassum muticum*. Additionally, Ferreres et al. [69], reviewed by de Sousa et al. [70], highlighted that *G. nodicaulis* and *E. selaginoides*, collected in West Portugal, produced also different phlorotannins belonging to eckol and fucophloroethol types, not observed in this present study.

### 3.4. Do Chemical Characteristics Could Help to Understand the Distribution of the Five Species along Brittany Rockyshores?

The distribution of Sargassaceae populations in rockpools results from a balance between (1) the capacity of the species to survive to a variable environment—the most changing zones being in the upper shore [71]—and the species needing to be tolerant regarding temperature and salinity and (2) their capacity to settle on a substrate yet colonized by competitors. *Cystoseira humilis* must be particularly resistant to changing environments so it can colonize the upper shore. In addition, only few species have such an ability, as suggested by the low species richness of its pools (equal to 7, see Figure 1), and by the low proportion of substrate covered by living organisms (Jégou, pers. obs.). Thus, the competition is low, and the recruits of *C. humilis* have much space to settle, which permits high densities within populations. Small length of individuals could be regarded as an adaptation of this species to shallow pools, or to pools in which depth can become very low due to evaporation in summer [13]. At the very opposite, *E. selaginoides* cannot face such intense environmental changes, and thus it cannot develop on mid and upper pools. However, it can grow on lower tide pools, but because the competition for the substrate is strong (high species richness up to 35, see Figure 1, Table 1), only low densities occur on the field. The high depth occurring more frequently in lower rock pools (in our survey, from 30 cm to more than 1 m) let the individuals grow taller than anywhere else. *Gongolaria baccata* turns out to follow this tendency; however, it has greater ecological amplitude, allowing its presence in mid pools. *Gongolaria nodicaulis* and *C. fœniculacea* can be regarded as intermediate species. Living in mid tidepools implicates a slightly higher variability of the water parameters than in lower pools, and a lower intensity of competition. Because these mid pools are frequently less deep than in the lower intertidal, this would lead to the occurrence of medium-sized individuals with an intermediate population density.

Throughout the survey, seasonality was, at first sight, not a typical feature within the five species. Only three of them, *C. fœniculacea*, *G. nodicaulis* and *E. selaginoides* showed unambiguous seasonal patterns, each species being characterized by a cycle in three phases: (1) active growth via the production of primary, secondary, tertiary (…) axes leading in (2) the development of mature receptacles, and after the release of the gametes, a loss of secondary axes that precedes (3) a dormancy period. This typical growth process has been known since Sauvageau [72] and Roberts [73,74,75] described it fairly. However, a time shift in these phases was observed between species. For example, *C. fœniculacea* and *C. nodicaulis* began dormancy in the middle of summer, while *E. selaginoides* was in its full maturity period (Table 1). Such an evident phenology could not be retrieved for *G. baccata* and *C. humilis* (Table 1). *Cystoseira humilis* was mature between spring and summer, but maturity can be observed all year long, depending on the dynamism of the population. Moreover, in Portugal, the reproduction period seems to be summer [76]. Reproduction period(s) must be under the influence of environmental parameters. Regarding *G. baccata*, it was not possible to determine seasonal patterns. Contrary to Le Lann et al. [19,77], we cannot deduce a unique reproductive period (Table 1) because all observed thalli were mature, all along the survey. However, the state of the algae differed throughout the seasons, even if it cannot be deduced from the results. It was obvious that during some periods, notably around summer, the number of receptacles was drastically reduced (Jégou, pers. obs.). A supplementary study on the evolution of the number of receptacles would probably highlight a seasonal pattern in the reproduction of *G. baccata*.

## 4. Materials and Methods

### 4.1. Native Canopy-Forming Sargassaceae Species and Sampled Rockpools

The correct identification of the specimens was here ensured using characteristic morphological and chemical features (see [78] for details about the criteria). In Brittany, the sites where the five species can be observed altogether are very rare because they have to fit some conditions, such as a sheltered site and an extended rocky intertidal zone. We chose to set up our experiment at Saint-Pierre (Penmarc’h, Brittany, France; 47°48′03″ N; 4°22′39.0″ W), where the slope of the shore is so low that the intertidal zone goes beyond 400 m in direction to the sea. The location of the five species was checked on the field using a Magellan™ Triton 200 GPS system, with an approximate 1 m precision. These tracks were deployed with a 60 m Stanley™ long tape rule. The presence of the Sargassaceae species was investigated using a 0.5 m × 0.5 m quadrat. For example, a 40 m track consisted of the observation of 80 aligned quadrats. For each species, the presence of individuals and their precise location on the intertidal zone were noticed. For statistical analyses purposes, we converted the location of all the individuals (latitude and longitude) into a one-dimensional position on the sea shore. We also evaluated the macroalgal species richness along the transect. This operation was repeated five times, from spring 2010 to spring 2011 (regarded as spring 2 to spring 3, respectively), to ensure the validity of the results, as suggested by Underwood [79]. Seasonal data were compiled to give the distribution of each species on Figure 1.

### 4.2. Extraction and Analysis of Pigments

**Sampling.** The five species were sampled on May 2011 and regarded as spring 3. We sampled three individuals without epiphytes per species and along the distribution of each species along the shore in order to take in consideration the large or the narrow distribution of species along the shore. The different pools were selected on the shore on the basis of similar characteristics, especially regarding depth (approximately 20 cm). Just after sampling, the thalli were thoroughly rinsed with deionized water, freeze-dried and stored in the dark at −20 °C until extraction.

**Extraction.** Algal samples (apical parts) were ground using liquid nitrogen with a mortar and a pestle. Then, 50 mg of the powdered alga was subsequently extracted twice with 1 mL of a mixture of acetone/water (90:10, *v*/*v*), at 4 °C and under agitation. The first extraction lasted 30 min, the second 12 h. The two extracts were then pooled, centrifuged at 5000 rpm during 5 min. The resulting supernatants were combined and then filtered using a 0.45 µm Nylon membrane (Millipore, Guyancourt, France). Prior to injection in the HPLC system, 150 µL of the filtered extract were mixed with 50 µL of buffer (ammonium acetate aqueous solution at 0.5 M, pH 7.2).

**HPLC analysis.** These samples were analyzed using an HPLC system using UV-Visible detection. For this purpose, a Waters HPLC system (Waters, Guyancourt, France) equipped with a Waters 717 Plus autosampler, a Water 600 Controller pump and a Photodiode Array Detector was used. The wavelength range of the detector was set at 210–700 nm. The pigments were separated following the method described by Wright et al. [80] modified by Bidigare et al. [81]. Each analysis included the injection of 10 µL of a sample. Pigments were separated using a Zorbax Eclipse XDB-C18 column (4.6×150 mm; 5 µm; Agilent Technologies, Les Ulis, France) equipped with a C18 guard cartridge (SecurityGuard, Phenomenex, Le Pecq, France) and maintained at 40 °C. A ternary mobile phase was used: eluent A was constituted by 80% methanol, 20% ammonium acetate buffer at pH 7.2, and BHT at 0.1 g/L; eluent B was constituted by 87.5% acetonitrile, 12.5% water, and BHT at 0.1 g/L; eluent C was pure ethyl acetate. The gradient of elution is indicated in Table S3 (Supplementary Material). Chlorophyll *a*, chlorophyll *c2*, fucoxanthin, violaxanthin, zeaxanthin and β-carotene (DHI, Hørsholm, Denmark) were used as standards.

### 4.3. Extraction of Phlorotannins and Determination of Phenolic Content

Samples were collected from spring 2009 (=spring 1) to summer 2010 (=summer 2) in order to follow the variability of phenolic content during six seasons.

**Extraction.** For each sample (3 individuals per species), phenolic compounds were extracted twice, successively from 200 mg of powdered algae with a methanol/water (1:1) mixture during 2 h, at 40 °C in the dark. The two extracts were pooled and semi-purified; methanol was evaporated and the volume of the resulting crude extract was set to 10 mL of aqueous solution. Then 5 mL of ethyl acetate was added and the resulting 15 mL was mixed and centrifuged (5000 rpm, 4 °C). The organic phase was isolated while the remaining aqueous phase was re-extracted once with 5 mL ethyl acetate. The two organic phases were combined and the solvent was removed using a rotary evaporator. The organic molecules were dissolved in 10 mL of water (containing less than 1% ethanol for a better solubility).

**Folin–Ciocalteu Assay.** The phenolic content of the semi-purified fraction was evaluated using the Folin–Ciocalteu assay slightly adapted from previous studies [17,82] and modified by Le Lann et al. [83]. Briefly, 100 mL of semi-purified fraction was mixed with 50 mL of Folin–Ciocalteu reagent, 200 mL of Na_2_CO_3_ (15%) and 650 mL of distilled water. This mixture was heated during 20 min at 70 °C and put on ice for 10 min to stop the reaction. The absorbance was measured at 750 nm. Standard phloroglucinol solutions were also submitted to this assay to obtain a calibration curve. The results were expressed as eq. phloroglucinol content in % DW.

### 4.4. NMR Analyses of Phlorotannins

Purification steps. Different purification steps of the crude extract were followed: three washings using dichloromethane (DCM), a precipitation step using ethanol following by another precipitation step using acetone, and a liquid–liquid water:ethyl acetate (EA) extraction was performed with the phenolic compounds concentrated in the EA phase, as described by several authors [41,44,55,56]. The last EA phase, containing the phenolic compounds, was recovered for further analyses.

**Fractionation on a silica column.** A 50 g silica column (Normal Phase Silica, 0.63–0.200 mm, Merck, Fontenay sous Bois, France) was packed in ethyl acetate. The sample was deposited at the top of the column, and three fractions were recovered by elution with (1) ethyl acetate, (2) ethanol and (3) methanol. The ethyl acetate fraction was further analyzed using 2D NMR analysis in the aim to determine the structure of phlorotannins.

**NMR analysis.** The various crude extracts and fractions were monitored by ^1^H 1D NMR analysis on a BRUKER Avance 400 MHz spectrometer (Bruker, Wissembourg, France) with a tunable triple resonance multi-nucleus probe (acquisition performed at 25 °C). For each species, the most interesting EA fraction was characterized in 2D ^1^H-^13^C NMR by heteronuclear correlation sequence on multiple bonds (HMBC) on a BRUKER Avance 500 Mhz spectrometer equipped with a cryoprobe. The HMBC sequence allows the determination of correlations between ^1^H and ^13^C at more than 2 or 3 bonds. The interest here was to show the proximity between aromatic protons compatible with phlorotannins (5.7 ppm ≤ δ ^1^H ≤ 6.5 ppm) with the carbons of the phenolic rings, and especially those involved in the linkage between phloroglucinol units, which allowed the determination of the major types of phlorotannins in each species [63].

### 4.5. Statistical Analyses

All analyses were performed using the R statistical software [84], with a type I error level α = 0.05. Considering the study of the distribution across the shore, we evaluated the differences between the positions of the species. When homogeneity of variances was rejected (Fligner-Killeen’s test), even after the use of common transformations of data, as proposed by Underwood [79], we used a Kruskal–Wallis test, and an associated multiple comparison test using the package “pgirmess” [85]. We investigated the effects of the factors “species” and “tidal height” on pigment and phenolic contents, using two-way ANOVA, and differences were highlighted by Tukey’s HSD test. For this purpose, we checked the required assumptions of normality (using Shapiro–Wilk test) and homoscedasticity (Levene’s test). Correlations between variables were assessed using Pearson’s method.

## 5. Conclusions

On the intertidal zone of the rocky shores of Brittany, *Cystoseira*, *Ericaria* and *Gongolaria* species can be under the control of analogue parameters to the ones determining the macroalgal assemblages on the emerged substrata. Considering phlorotannins, our study highlights differences in content between species, without being possible to directly link phlorotannin content and distribution on the foreshore. Moreover, each species was characterized by its own seasonal dynamics of phlorotannin levels. Further investigations, extending the monitoring over several years and also measuring the exuded phenolic compounds in the rockpools could shed some light on the ecological role of phlorotannins. From a qualitative point of view, numerous polymers make up the mixture of phlorotannins in all species, except *E. selaginoides* which produces the monomer phloroglucinol. Thus, this species is a very good candidate for ecophysiological or functional genomics approaches.

## Figures and Tables

**Table 1:** Phenological variables from the five species of concern: the upper species *Cystoseira humilis*, the two median species *C. fœniculacea* and *Gongolaria nodicaulis* and the two lower species *G. baccata* and *Ericaria selaginoides*. Density represents the number of individuals counted within a surface of 0.25 m^2^. Variables were monitored from winter 2009 (=winter 1) to summer 2010 (=summer 2). W: winter, Sp: spring, Su: summer, A: autumn.

		Position of the Rockpool along the Intertidal Zone				
		Upper Position	Median Position			Lower Position
		*C. humilis*	*C. fœniculacea*	*G. nodicaulis*	*G. baccata*	*E. selaginoides*
Density (ind/0.25 m^2^)	Min.	2 (W1)	1.33 (W1)	1 (W1)	1	1 (Sp1/A1/W1)
	Max.	8.25 (Su2)	5.33 (Su2)	4.33 (Su1)	2 (A1)	1.33 (Su1)
Percentage cover	Min.	12.5% (W1)	17.5% (Sp1)	13.33% (W1)	26.67% (W1)	10% (Sp2)
	Max.	60% (A1)	56.67% (A1/W1)	50% (Su2)	90% (Sp1)	91.67% (Su1)
Maturity	Min.	16% (W1)	0% (A)	22.36% (Su1)	100% all year round	0% (W/Sp)
	Max.	100% (Sp/Su2)	100% (Sp)	100% (W/Sp1)		100% (Su/A)
Mean length of individuals	Min.	21.63 cm (Su2)	15.68 cm (Su2)	22.36 cm (Su1)	53.67 cm (Su2)	25.66 cm (W2)
	Max.	33.05 cm (W2)	48.40 cm (W1)	58.33 cm (Sp1)	102.33 cm (Sp1)	56.67 cm (Sp1/A1)
Number of macroalgal taxa in the rockpool		7	7–13	7–13	10–35	13–35

**Table 2:** Types of phlorotannins observed in our study in the five species of *Cystoseira*, *Gongolaria* and *Ericaria*. Examples of phlorotannin structures are also given. Mean total phenolic content (TPC) was calculated from spring 1 to summer 2.

	Mean TPC (% DW)	Type of Phlorotannins (from 2D NMR Results)			
		Phloroglucinol	Fucol	Phlorethol	Fucophlorethol
*C. humilis*	0.19 ± 0.09	Yes	No	Yes	No
*C. fœniculacea*	0.13 ± 0.06	/	/	/	/
*G. nodicaulis*	0.25 ± 0.16	No	Yes	Yes	Yes
*G. baccata*	0.21 ± 0.10	Traces	Yes	Yes	Yes
*E. selaginoides*	0.42 ± 0.21	Yes	No	No	No
Examples of chemical structures of phlorotannins		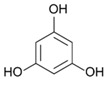	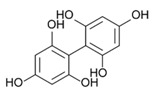	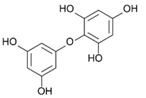	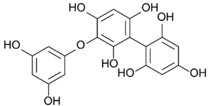

## Data Availability

The data presented in this study are available on request from the corresponding author.

## Supplementary Materials

The following are available online at https://www.mdpi.com/article/10.3390/md19090504/s1, Table S1: Results of the two-way ANOVA (pigment levels as a function of the species and tidal height of settlement), Df: degree of freedom, Significant results are highlighted., Table S2: Correlation between pigment levels according to Pearson’s test; all indicated values are significant (*p* < 0.001), n.s.: no significant correlation (β-car: β-carotene; Chl: chlorophyll; Fuco: fucoxanthin; Viola: violaxanthin and Zea: zeaxanthin)., Table S3: Solvent gradient used during HPLC analysis of pigments in *Cystoseira*, *Ericaria* and *Gongolaria* species.
