# Supplementary material for: Phlorotannin and Pigment Content of Native Canopy-Forming Sargassaceae Species Living in Intertidal Rockpools in Brittany (France): Any Relationship with Their Vertical Distribution and Phenology?
Source: Mar Drugs. 2021 Sep 4;19(9):504. doi: 10.3390/md19090504 (PMC8469379; doi:10.3390/md19090504)
Supplement: Supplementary file 1 [file marinedrugs-19-00504-s001.zip › marinedrugs-1342469-supplementary.pdf]

Supplementary material for Jégou et al. "**Phlorotannin and pigment content of native canopy-forming Sargassaceae species living in intertidal rockpools in Brittany (France): any relationship with their vertical distribution and phenology?**"

Table S1. Results of the two-way ANOVA (pigment levels as a function of the species and tidal height of settlement), Df: degree of freedom, Significant results are highlighted.

	**Chlorophyll *a***			**Chlorophyll *c2***			**Fucoxanthin**		
	Df	F	p	Df	F	p	Df	F	p
**Species**	4	11.60	**<0.001**	4	6.73	**0.002**	4	9.09	**<0.001**
**Tidal height**	2	9.91	**0.002**	2	3.24	0.065	2	16.66	**<0.001**
**Interaction**	1	0.08	0.778	1	1.25	0.280	1	2.46	0.136

	**β-carotene**			**Violaxanthin**			**Zeaxanthin**		
	Df	F	p	Df	F	p	Df	F	p
**Species**	4	13.05	**<0.001**	4	5.20	**0.007**	4	15.53	**<0.001**
**Tidal height**	2	2.36	0.126	2	3.64	**0.049**	2	0.47	0.634
**Interaction**	1	0.13	0.724	1	0.042	0.840	1	1.31	0.269

Table S2. Correlation between pigment levels according to Pearson's test; all indicated values are significant (p<0.001), n.s.: no significant correlation (β-car: β-carotene; Chl: chlorophyll; Fuco: fucoxanthin; Viola: violaxanthin and Zea: zeaxanthin).

	β-car	Chl *a*	Chl *c2*	Fuco	Viola	Zea
β-car	-	0.74	0.81	0.71	0.87	n.s.
Chl *a*			0.80	0.90	0.84	n.s.
Chl *c2*				0.85	0.88	n.s.
Fuco					0.85	n.s.
Viola						n.s.
Zea						-

Table S3. Solvent gradient used during HPLC analysis of pigments in *Cystoseira*, *Ericaria* and *Gongolaria* species

Time (min)	Flow (mL/min)	% A	% B	% C	Event
0	1	90	10	0	Injection
1	1	0	100	0	Linear gradient
11	1	0	78	22	Linear gradient
27.5	1	0	10	90	Linear gradient
29	1	0	100	0	Linear gradient
36	1	90	10	0	Equilibration
